# Dual Relationship Between Stromal Cells and Immune Cells in the Tumor Microenvironment

**DOI:** 10.3389/fimmu.2022.864739

**Published:** 2022-04-06

**Authors:** Jeong-Yeon Mun, Sun-Hee Leem, Jun Ho Lee, Hyuk Soon Kim

**Affiliations:** ^1^ Department of Biomedical Sciences, College of Natural Science, Dong-A University, Busan, South Korea; ^2^ Department of Health Sciences, The Graduate School of Dong-A University, Busan, South Korea; ^3^ College of Korean Medicine, Woosuk University, Jeonju, South Korea

**Keywords:** tumor microenvironment, immune cells, stromal cells, cancer-associated fibroblast (CAF), tumor endothelial cell, cancer-associated adipocyte, T cell, NK cell

## Abstract

The tumor microenvironment (TME) plays a critical role in tumorigenesis and is comprised of different components, including tumor cells, stromal cells, and immune cells. Among them, the relationship between each mediator involved in the construction of the TME can be understood by focusing on the secreting or expressing factors from each cells. Therefore, understanding the various interactions between each cellular component of the TME is necessary for precise therapeutic approaches. In carcinoma, stromal cells are well known to influence extracellular matrix (ECM) formation and tumor progression through multiple mediators. Immune cells respond to tumor cells by causing cytotoxicity or inflammatory responses. However, they are involved in tumor escape through immunoregulatory mechanisms. In general, anti-cancer therapy has mainly been focused on cancer cells themselves or the interactions between cancer cells and specific cell components. However, cancer cells directly or indirectly influence other TME partners, and members such as stromal cells and immune cells also participate in TME organization through their mutual communication. In this review, we summarized the relationship between stromal cells and immune cells in the TME and discussed the positive and negative relationships from the point of view of tumor development for use in research applications and therapeutic strategies.

## Introduction

The tumor microenvironment (TME), a highly heterogeneous environment composed of many different types of cells and many molecules produced or released by tumor cells, stromal cells, and immune cells, is now widely recognized (1). The TME is composed of cellular components such as cancer-associated fibroblasts (CAFs), tumor-endothelial cells (TECs), cancer-associated adipocytes (CAAs), mesenchymal stem cells (MSCs), T cells, B cells, natural killer (NK) cells, and tumor-associated macrophages (TAMs) (2). In addition, the TME is rich in hypoxic, acidic, and immune/inflammatory cells known to play important roles in tumor development, growth, progression, and resistance to treatment (3–6). In the past, cancer therapies were designed to target cancer cells directly. Recently, they are designed to destroy networks formed by tumors. Immunotherapy, in addition to surgery, chemotherapy, and radiation therapy, has emerged as a breakthrough treatment modality for cancer patients (7). Besides therapies that directly target tumor cells, promising therapies targeting stromal cells present in the TME are also attracting attention. Targeting immunomodulatory pathways in the TME is considered a central step in cancer treatment (8–10). Indeed, the TME is currently in the spotlight as a new target for cancer therapeutics with many ongoing studies.

Treatments for cancer patients include surgical resection and chemotherapy or radiation therapy (11). With recent advances in onco-immunological studies, the use of immune checkpoint inhibitors (ICIs) taking advantage of the antitumor activity within the TME is considered as an effective therapeutic modality for cancer patients. Among them, inhibitors of the cytotoxic T lymphocyte antigen-4 (CTLA-4) blockade have shown remarkable efficacy in clinical trials. Both ipilimumab and tremelimumab are human antibodies to CTLA-4 (12, 13). In recent studies, both drugs have achieved considerable clinical success for patients with advanced malignant melanoma. A phase 3 study has shown that ipilimumab, a CTLA-4 blockade, can significantly improve the overall survival of melanoma patients. Based on results of these studies, ipilimumab, a T-cell potentiator, is considered useful as a treatment for advanced melanoma patients (14, 15). In addition, it has been suggested that PD-1/PD-L1 expression plays an important role in immune evasion by cells other than tumor cells present in the TME. Nivolumab is an ICI targeting PD-1/PD-L1. It is being used to treat melanoma, non-small cell lung cancer, kidney cancer, hepatocellular carcinoma, urinary tract cancer, gastric cancer, and triple-negative breast cancer (TNBC) (16–18). In addition to immunotherapy-based ipilimumab and nivolumab, a variety of FDA-approved ICI treatments such as cemiplimab and pembrolizumab are also being actively implemented for cancer treatment (19).

The expression of a modified T-cell receptor or chimeric antigen receptor (CAR) to enhance antigen specificity can be engineered using a patient’s own T cells. Thus, it is possible to efficiently generate tumor-targeting T cells by solving the fundamental problem without requiring the patient to activate *de novo* T cells (20, 21). Clinical trials conducted on patients with acute lymphocytic leukemia have shown a complete recovery in up to 92% of patients with very meaningful results (20). CAR-T cell therapy is being actively implemented for treating large B-cell lymphoma and B-cell precursor acute lymphoblastic leukemia (22, 23). Although this CAR-T cell therapy has achieved clinical success in some hematologic cancer patients (24), it has two main problems that need improvement. First, it is very difficult to obtain enough T cells from cancer patients to isolate CAR-T cells because of lymphocytopenia due to prior treatments. Second, there is not enough time to implement CAR-T cell therapy for rapidly advancing cancers (25). Tumor-infiltrating lymphocyte (TIL) therapy can be proposed as a form of therapy to solve these problems. TIL therapy is a treatment involves removing T cells infiltrating a patient’s tumor, proliferating them to a large amount in the laboratory, and injecting them back into the patient to help the patient’s immune system kill cancer cells (26). It has the advantage of being able to locate and destroy the patient’s tumor directly because it secures T cells that have already penetrated the patient’s tumor and reinjects them into the patient. Activated NK cells can also directly lyse tumor cells by releasing cytotoxic granules (including perforin and granzymes) in a manner similar to that of activated cytotoxic T cells. Since NK cells can eliminate tumors, immunotherapy based on NK cells has been developed. It is currently being used strategically. In addition, CAR-NK cells, like CAR-T cells, are genetically modified to express CARs that can recognize specific antigens that are characteristically overexpressed by target cells. Preclinical studies of CAR-NK cells have been performed on hematologic cancers and some solid cancers (27). These CAR-NK cells, along with T cell-based therapies, could be proposed as an improved therapy for solid tumors.

Strategies using drugs that can specifically target stromal cells within the TME have been proposed to further enhance patient survival and therapeutic effects. Therapeutic drugs that target stromal cells within the TME are under investigation (28). These stromal cells are important components of the TME as they express specific markers that can be targeted for tumor treatment (29). Of note, treatment with microsomal prostaglandin E synthase-1 inhibitor compound III targeting CAF-derived prostaglandin E2 (PGE2) can reduce tumor growth, suppress CAF migration/infiltration, and increase M1 macrophage ratio in neuroblastoma tumor studies (30). Furthermore, studies using a mouse model of cholangiocarcinoma induction have confirmed that navitoclax (BCl-2 inhibitor) treatment can induce CAF apoptosis, reduce the expression of tenascin C, and suppress tumor growth. This suggests that navitoclax may strategically target and destroy CAFs within TMEs to attack tumors (31). As an inhibitor of fibroblast growth factor receptors (FGFR), PD173704 can reduce the growth of both CAFs and endothelial cells (ECs), thereby inhibiting stromal cell-mediated FGFR pathway in a co-cultured environment of head and neck squamous cell carcinoma (HNSCC) cells. These inhibitor molecules can inhibit tumor cell growth, thus playing a crucial role in tumor-matrix interaction. They have been proposed as potential therapeutic agents for HNSCC (32). Phosphodiesterase (PDE), a class of enzymes that can hydrolyze cyclic adenosine monophosphate (cAMP) and cyclic guanosine monophosphate (cGMP), are composed of 11 different subtypes. A lung cancer study has shown that phosphodiesterase-4 (PDE4) as a type of PDE can promote lung cancer proliferation and angiogenesis by having a crosstalk with hypoxia-inducible transcription factors (HIFs) factor (33). CC-5079 is an analog of these PDE4 inhibitors. It can inhibit the proliferation and migration of fibroblasts, bladder cells, and EC cells, stimulate mitogen-activated protein kinase phosphatase 1 (MKP1) expression, and inhibit micro-angiogenesis through its upregulation. It has been demonstrated that CC-5079 is a candidate for treating colon cancer by targeting ECs within the TME (34). However, further studies on the detailed mechanism of action of CC-5079 are needed. Although the TME component targeting therapy such as immune cell targeting therapy and stromal targeting therapy has been applied to treat cancer patients in various studies mentioned above, it has some limitations because the TME is heterogeneous, showing different characteristics in each patient (29).

Although the role of the TME in cancer processes has been extensively investigated, its contribution to the crosstalk between stromal cells and immune cells, which are components of the TME based on the tumor, is only partially known. Previous therapeutic approaches have focused on cancer cells and established relationships between cancer cells and immune cells or stromal cells. Based on an understanding of the relationship, it is more effective to consider treatment methods in a direction that can restore the relationship between stromal cells and cancer cells and the self-dependent role in the TME by understanding the relationship between cells in the TME. Communication between TME constituent cells should be understood and therapeutically targeted. Characteristic parts of cells must be considered. In this respect, unlike cancer cells, the somatic cells that make up our body are controllable. The need to find ways to overcome cancer treatment by properly regulating the communication between immune cells has emerged. In particular, exploring interactions and intercellular relationships in the TME will improve our understanding of cancer treatment (35, 36). Therefore, in this review, we described and summarized interactions, roles, and importance of the relationship between stromal cells and immune cells within the TME.

## The Pro-Tumorigenic Effect of the Relationship Between Stromal Cells and Immune Cells in the TME

### CAFs Interact With Myeloid Cell-Derived Immune Cells in the TME to Enhance Tumorigenesis and Immune Evasion

CAFs are the most dominant cell type in the TME. They are known to crosstalk with immune cells (37). CAFs may play a pivotal role in tumor development and survival as they are involved in TME composition and participate in mechanisms that promote tumor growth and invasion and subvert defense system (38–40). Recently, studies have attempted to clarify this point. CAFs can secrete components such as cytokines and chemokines known to be continuously activated in the TME through various signaling mechanisms and function as primary immunosuppressive mediators (41, 42). CAF-derived cytokines and chemokines are attracting attention not only for their roles in tumor progression, but also for their ability to regulate the recruitment and function of immune cells.

TAMs are macrophages that participate in the formation of the TME by producing cytokines, chemokines, and growth factors. TAMs are divided into two types, M1 and M2 macrophages (43, 44). Characteristically, M1 macrophages produce large amounts of pro-inflammatory cytokines and regulate the Th1 antitumor immune response, whereas M2 macrophages play an important role in tumor progression. Gokyavuz et al. have shown that CAFs can secrete monocyte chemotactic protein-1 (MCP-1) and stromal cell-derived factor-1 (SDF-1) and effectively recruit monocytes (45). MCP-1 is a chemokine that contributes to the recruitment of monocytes to the site of an inflammatory response. It is expressed in a variety of cancer types such as prostate and ovarian cancers (46, 47). SDF-1 is expressed in stromal fibroblasts in organs including the brain, breast, and lung. It is involved in cancer survival, proliferation, and metastasis (48, 49). Specifically, monocytes recruited by CAFs *via* MCP-1 and SDF-1 can reduce the secretion of pro-inflammatory cytokine interleukin (IL)-12 and increase the production of anti-inflammatory cytokine IL-10. CAF-educated monocytes can increase the motility and invasiveness of breast cancer cells and the expression of epithelial-mesenchymal transition (EMT)-related genes and vimentin proteins, eventually exerting their immunosuppressive role in breast cancer (45). In prostate cancer, CAFs can also promote the differentiation to the M2 macrophage phenotype *via* SDF-1. Analysis of patients with prostate cancer has confirmed a clear increase in the M2/M1 macrophage ratio, which is correlated with clinical prognosis. Thus, CAFs and M2-polarized macrophages actively contribute to the promotion of the invasive ability of prostate cancer cells. They are correlated with the aggressiveness of cancer *via* the infiltration of M2 macrophage (50). Chemokine (C-C motif) ligand 2 (CCL2; MCP-1) secreted by CAF can induce blood monocyte recruitment and differentiate into TAMs in breast cancer (51). Additional research has confirmed that the increase in monocyte migration to breast tumor spheroids is associated with CAF-derived CCL2 and that the CCR2A/2B pathway and the CCL2 receptor play important roles in monocyte recruitment (52). These findings suggest that CAF-derived factors can affect a wide range of processes, leading to monocyte recruitment and M2 macrophage differentiation.

Among various cancer types, pancreatic cancer has a low response to immunotherapy. Thus, it is necessary to understand the antitumor immune response in pancreatic stromal cells. Stromal cells in the pancreatic cancer microenvironment can generate numerous factors that support the growth and survival of tumor cells. They are being studied to increase the understanding and relevance of immune cells (53). Pancreatic stellate cells (PSCs) are resident cells in the pancreas under quiescent conditions. They are isolated from the human pancreas as fibroblast-like cells (54). These cells display some characteristics of activated myofibroblasts such as α-smooth muscle actin (α-SMA) expression and ECM proteins synthesis (55). Much evidence has confirmed the importance of PSCs in pancreatic ductal adenocarcinoma (PDAC) development. Importantly, myeloid-derived suppressor cells (MDSCs) are identified as immature myeloid cells that can induce immunosuppression, mediate multiple signaling pathways, and interact with immune cells and mediators (56, 57). It has been shown that MDSCs can promote cancer progression, angiogenesis, and metastasis and disrupt the efficacy of therapeutic agents (58, 59). In particular, the number of MDSCs is highly correlated with the staging of pancreatic cancer patients, indicating that an increase in MDSC levels could be an indicator of disease progression (60). Thus, MDSCs could be targeted in the treatment of PDAC patients. Mace et al. have reported that soluble factor IL-6 generated from patient PSCs with confirmed phenotypic α-smooth muscle actin and glial fibrillary acidic protein expression can promote the differentiation of MDSCs (61). PSCs in PDAC can modulate MDSC differentiation *via* IL-6 and STAT3 signal transduction pathways (61). Targeting PSCs in the TME by promoting the generation of immunosuppressive cells that suppress the innate or acquired immune response against pancreatic cancer may reduce MDSC levels, thereby enhancing the effectiveness of immunotherapy. Dendritic cells (DCs) represent immune cells that link innate and adaptive immunity. They are derived from hematopoietic bone marrow precursor cells (62). DCs are essential antigen-presenting cells (APCs) that can induce the activation of naïve T cells. Levels of peripheral blood DCs are decreased in cancer patients than in normal controls (63–65). By expressing high levels of immunoregulatory cytokines, they can induce the differentiation of regulatory T cells (Tregs) and help tumor cells evade the immune response. Cheng et al. have shown that CAF-derived IL-6 can induce the activation of the STAT3 pathway, leading to immunosuppressive cell types (66). Especially, STAT3 activation by CAF-derived IL-6 plays a pivotal role in indoleamine-2,3-dioxygenase (IDO) production and induced regulatory DC recruitment (66). Cheng et al. have evaluated CAF-regulated neutrophil function and the activation of hepatocellular carcinoma (HCC) *via* the IL-6/STAT3/PD-L1 signaling cascade (67). They found that IL-6 derived from CAFs could recruit PD-L1^+^ neutrophils and impair T-cell function *via* PD1/PD-L1 signaling (67). Cho et al. have reported that IL-6 and granulocyte-macrophage colony-stimulating factor (GM-CSF) released from cancer cell-activated CAFs through co-culture of monocytes and CAFs can increase TAM infiltration and metastasis and direct monocytes to differentiate into M2 TAMs (68). Thus, the development of inhibitors or neutralizing antibodies targeting IL-6, IDO, GM-CSF, and STAT3 may lead to a new cancer immunotherapeutic approach that can induce tumor immune evasion of CAFs *via* the cell network in the TME. In the TME, secreted chemokines including IL-8 play important roles in tumorigenesis. These chemokines could be secreted by CAF (69). According to a study on colorectal cancer-derived CAFs isolated from human colorectal cancer tissue, the CAF-derived IL-8 can be secreted to attract monocytes and promote polarization from anti-tumorigenic/pro-inflammatory M1 macrophages to pro-tumorigenic/anti-inflammatory M2 (70). These findings indicate that CAFs can also inhibit NK cell function and promote CRC cell progression by increasing TAM infiltration.

### CAFs Interact With Lymphocytes in the TME to Enhance Tumorigenesis and Progression

Transforming growth factor-β (TGF-β) is a representative cytokine secreted from CAF with a strong immunomodulatory function. It is involved in the suppression of immune responses. It also plays an important role in tumor initiation, invasion, and metastasis (71–73). CAFs-derived TGF-β can regulate various types of immune cells through the paracrine signaling pathway with growth factors and cytokines. TGF-β can suppress IL-2 production and T cell proliferation. It plays an important role in CD4^+^CD25^+^ Treg production and function (74). Furthermore, it has been proposed that transcriptional inactivation of TGF-β by suppressing T-box expressed in T cells (T-bet) and GATA-binding protein 3 (GATA-3) expression can control the differentiation of murine CD4^+^ T cells (75, 76). According to immunological studies conducted in HCC cells, IL-10, TGF-β, and IL-4 secreted by stromal cells in the TME can increase polarization to M2 macrophages. It is known that M2 macrophages can enhance tissue remodeling, angiogenesis, tumor progression, and ultimately IL-10, increasing PD-L1 and human leukocyte antigen (HLA)-DR expression to induce immunosuppression (77–79).

Since stromal cells in the TME express cytokines and immunological factors, it is critical to consider stromal cells to rescue tumor-reactive CD8^+^ T cells from immune evasion mechanisms (80). According to a study by Lakins et al., CAF-educated T cells can induce death of T cells by PD-L2 and Fas ligand (FasL) engagement in the lung tumor stroma (81). These results indicate that CAFs can enhance tumor viability by inducing the dysfunction of encountered CD8^+^ T cells, suggesting that CAFs can directly contribute to the pro-tumor T cell immune response.

Wu et al. have investigated the heterogeneity of stromal cell population of TNBC patients using single-cell RNA sequencing technology and confirmed the existence of an inflammatory CAF (iCAF) subpopulation (82). These iCAFs showed upregulation of CXCL12 (SDF-1)-CXCR4 chemoattractant pathway genes and suggested a strong association of CD8^+^ T cell dysfunction with iCAF presence and exclusion (82). In breast cancer studies, four different CAF subsets of human breast cancer have been identified, among which the CAF subset 1 is characterized as immunosuppressive cells. CAF subset 1 can secrete CXCL12 to attract T cells, increase the survival of CD4^+^CD25^+^ T cells, and promote differentiation into CD25^high^FOXP3^high^ Treg cells *via* B7H3, CD73, and DPP4 (83, 84). These studies indicate that the CAF-S1 fibroblast subset contributes to immunosuppression in breast cancer. Takahashi et al. have shown that IL-6, CXCL8, tumor necrosis factor (TNF), and vascular endothelial growth factor (VEGF)-α are detected more in CAFs than in normal fibroblasts in the TME of HNSCC. These CAFs can modulate effector T cell function by expressing co-regulatory molecules B7H1 and B7DC and enhance T cell apoptosis and the induction of Treg cells (85). Thus, CAFs have been implicated in the proliferation of CD4^+^FOXP3^+^ Treg cells, tumor progression, angiogenesis, and metastasis (86, 87). These cells play an important role in angiogenesis, invasion, and metastasis. They contribute to the immunosuppressive process that promotes tumor evasion by the T cell network within the TME of HNSCC. Moreover, CAFs can release different factors including chemokines, cytokines, and growth factors that can promote immunosuppression through recruitment of immunosuppressive cells such as Tregs and myeloid cells, upregulation of immune checkpoint molecules on T cells, and regulation of T cell migration (88). Notably, in PDACs, T cells can be inhibited by tumor stroma, suggesting that CAF-derived factors may regulate T cell function and phenotype (89–91). Most T cells in the TME are exhausted, leading to cancer immune evasion. Restoring exhausted T cells appears to be an excellent strategy in cancer immunotherapeutic therapy (92). PD-1, a critical inhibitory receptor regulating T cell exhaustion, can attenuate its ability to clear cancer *via* high expression in T cells (91). Reversing this T cell exhaustion represents a major strategy for cancer treatment (93).

Candidate factors that can interfere with signal transduction between CAF and NK cells have been suggested as potential strategies for cancer treatments (94). NK cells are cells that can produce cytokines to communicate with other cells. They have the ability to kill tumor cells (95, 96). NK cell activation releases perforin, granzymes, inflammatory cytokines, and chemokines toward target cells (97). PGE2 is a major product of arachidonic metabolism. It is synthesized *via* cyclooxygenase-2 (COX2) and prostaglandin synthase pathways (98). According to Balsamo et al., PGE2 released by melanoma-derived fibroblasts can suppress the expression of NK receptors, perforins, and granzymes, meaning that they can interfere with the phenotype and function of NK cells (99, 100). PGE2 secreted from HCC-associated fibroblasts can inhibit the expression of NK receptors exhibiting immune function, including NKp30, NKp44, and NKG2D, thus impairing NK cell-mediated cytolytic activity (101). In addition, it has been confirmed that blocking the activity of PGE2 and IDO in activated HCC-related fibroblasts can restore the function of NK cells and promote the progression of HCC (102). Thus, metastatic melanoma-derived fibroblasts and HCC-associated fibroblasts can release PGE2 and IDO to affect NK cell function and exert strong immunosuppressive activity (99–101).

### TECs, CAAs, and MSCs Interact With Immune Cells in the TME to Induce Immune Evasion and Reduce Anti-Tumor Functions

Endothelial cells (ECs) are a major type of cells found inside the lining of blood vessels, lymph vessels, and heart. They fulfill many physiological processes in the body (103). ECs are included in stromal cells. They represent an important interface between tissue and blood (104). TECs found in most tumors can also form an essential vascular inner layer in tumors (105). TECs play an important role in orchestrating the TME. TECs are known to be particularly important for T cell recruitment and activation. Previous studies have shown that the interaction between T cells and ECs plays an important role in the regulation of the immune system during chronic inflammation (106). TECs in the tumor microenvironment are particularly relevant to circulating immune cells. They may influence anti-tumor cell immune responses. TECs are APCs with an inhibitory activity. They express MHC class II and PD-L1. They can impair the production of pro-inflammatory cytokines including IL-2, TNF-α, and IFN-γ in CD8^+^ T cells (107, 108). TECs are also known to play a critical role in tumor cell growth and invasion (109). Vascular cell adhesion molecule 1 (VCAM1) induction in endothelial cells can regulate tumor progression, provide angiogenic factors, promote neutrophil infiltration and tumor cell adhesion to the endothelium, and promote metastasis by sustaining vascular Notch1 signaling (110).

In gliomas, penetration of T cells into tumor tissue as TILs is extremely low. Although this feature is controversial for the correlation with TIL-induced tumor prognosis in glioma, it has a useful aspect in the evaluation of factors that reduce the presence of T cells in tumor tissues. Moreover, FasL is well-known as a pro-apoptotic cell surface protein that plays an important role in confirming T cell depletion in tumor tissues. This has been demonstrated by flow cytometric analysis, showing that FasL levels expressed in the endothelium of brain tumors are inversely correlated with the CD8^+^/CD4^+^ TIL ratio (111). That is, FasL expression indicates not only the CD8^+^/CD4^+^ TIL ratio, but also the tumor contribution by immune avoidance in brain tumors due to decreased T cell presence. In melanoma, it has been confirmed that TECs are APCs that express MHC class II and PD-L1 with an inhibitory activity. These TECs can inhibit the proliferation of CD8^+^ T cells *via* inhibitory cytokines including IL-10 and TGF-β. They can also attenuate the antitumor effect of antigen-specific CD8^+^ T cells. Experimental results have shown that TECs can induce immune responses of tumor antigen-specific CD8^+^ T cells through the PD-1/PD-L1 pathway and evade tumor immunity by regulating immunosuppressive CD4^+^ T cells in an antigen-specific manner (112). Common lymphatic endothelial and vascular endothelial receptor (CLEVER-1/stabilin-1) are also expressed on lymphatic vessels, high endothelial venules, and non-continuous endothelium (113). CLEVER-1/stabilin-1 identified in HCC endothelium can recruit FOXP3^+^ Treg cells (114). These findings make it possible to confirm the tumorigenic effect of TECs under the influence of various mechanisms and factors.

CAAs play a central role in tumorigenesis, tumor growth, and metastasis (115). CAAs can help cancer cells by storing energy as triacylglycerol and directly providing lipids. They can release pro-inflammatory cytokines such as IL-8, CCL2, VEGF, TGF-β, and cathepsin S that can recruit bone marrow cells to the TME, thus regulating the differentiation of M2/MDSC and promoting the angiogenesis process (116–119). A breast cancer study by Arendt et al. has shown that adipocytes in human and mouse breast tissues can activate and recruit macrophages *via* the CCL-2/IL-1β/CXCL12 signaling pathway (117). These activated macrophages are sufficient to promote angiogenesis and accelerate stromal vascularization and breast cancer formation. Understanding major interactions between immune cells and CAAs in the TME can be an effective way to improve the effectiveness of existing therapies. PD-L1 can regulate anti-tumor immunity as described above. It is the main target of checkpoint-blocking immunotherapy. Previous studies on breast cancer have shown that the contribution of PD-L1 expression to adipogenesis remains an issue that merits further investigation. During the adipogenesis process, the expression of PD-L1 in primary human adipose stromal cells and adipocytes is highly induced. It is known that PD-L1 expression in breast cancer adipocytes is significantly elevated and that adipocyte-derived PD-L1 can inhibit the activity of important antitumor functions of CD8^+^ T cells (120). Fatty acids (FAs) as CAA-derived metabolites can influence immune cell homeostasis and differentiation (121), leading to immune evasion and tumor progression. Based on this, FAs are necessary for promoting proper differentiation of neutrophils. They can potentially promote the function of tumor-associated neutrophils (TANs). Besides FAs, CAA-derived IL-8 can recruit neutrophils to the TME. In PDAC, neutrophils are recruited by CAA-derived IL-1β to promote tumor progression and further activate PSCs (122).

In the TME, MSCs are multipotent stromal stem cells found in most cancers that play a central role in cancer cell growth, invasion, and metastasis by interacting with the tumor and immune cells in the TME (123, 124). MSC-derived TGF-β can increase the frequency of Treg cells, protect breast cancer cells, and support the growth of breast cancer (125). These MSCs can induce IL-10^+^ regulatory B (Breg) cells involved in the production of immunosuppressive environments through SDF-1α and CXCR7 (126). IL-10^+^ Breg cells are a subset of immunosuppressive cells. The frequency or the function of Breg cells is involved in the tumorigenesis of some cancers (127–130). It has been shown that PD-L1 in stromal cells in the TME can be induced by TNF-α signals to promote the progression of colorectal cancer by suppressing the antitumor immune response of CD8^+^ T cells (131). Recently, research and development for antitumor treatments targeting CAAs has progressed rapidly. Because CAAs can interact with TME component cells in a complex cell network, they can be combined with a variety of therapies, including targeted or immunotherapies, which can selectively eliminate tumor-promoting CAAs.

### Immune Cells Contribute to Immune Suppression With Stromal Cells in the TME

In the TME formed by tumor cells, the interaction between immune cells and stromal cells plays an important role in both cancer progression and anticancer activity. Among various types of immune cells in the TME, neutrophils have received less attention than other immune cell types. IL-1β is a cytokine secreted by neutrophils that can activate ECs. Treatment with IL-1 receptor antagonists can reduce *in vitro* endothelial cell migration, where IL-1β secreted by Ly6G^+^ neutrophils can directly activate endothelial cells and MMP-9, thus effectively increasing metastasis capability (132, 133). This demonstrates the distinct metastatic role of Ly6G^+^ neutrophils and confirms that they are beneficial for promoting tumors (132, 134). These increases of MMPs not only contribute to local invasion and metastasis-related cascades, but also contribute to the intravascular invasion process (135–137). Further research is needed to determine the role of neutrophils in the TME of various cancer types.

Among various immune cells, TAMs exist in the vicinity of CAFs and constitute the most abundant innate immune cell type (138). TAMs are classified as pro-tumorigenic macrophages that can promote the development of malignant tumors and activate CAFs to aid in tumor progression (139, 140). Tokuda et al. have shown that osteopontin is a key molecule involved in cancer-CAF-TAM interactions and that increased osteopontin can promote malignant tumors (141). This characterizes the importance of cancer cell-TAM-CAF interactions in HCC. Lung cancer is developed in the region of major fibrosis. Particularly, idiopathic pulmonary fibrosis case has been reported to be associated with increased risk of lung cancer (142, 143). Wnt7a secreted by M2 macrophages in pulmonary fibrosis can interact with Frizzled-1 to activate Wnt/β-catenin signaling and promote differentiation of MSCs into myofibroblasts. In this way, myofibroblasts activated in idiopathic pulmonary fibrosis can lead to accumulation of ECM, resulting in an acute exacerbation of lung disease such as lung cancer (144, 145). In addition, TAMs can regulate communication with cancer cells and other stromal cells in the TME through nanovesicle secretion, which carries various molecules, including microRNAs (146). Exosomes containing microRNAs can affect cellular processes and promote tumor progression and angiogenesis. Macrophage-derived exosomes containing miR-155-5p and miR-221-5p can be transmitted to ECs *via* an E2F Transcription Factor 1 (E2F1)-dependent manner, thus promoting EC proliferation and PDAC growth (147, 148).

Human decidual NK cells are CD56^superbright^CD16^-^ that can increase tumor growth by angiogenic activity *via* the production of VEGF, placental growth factor, and IL-8 (149). In non-small cell lung cancer patients, pro-angiogenic factors such as VEGF and placental growth factor are released from NK cells and defined NK cell subsets to promote human umbilical vein endothelial cell migration and capillary-like structure formation (150, 151). Mast cells in the TME can regulate adaptive immunity to tumors. Recent studies have shown that the infiltration of mast cells into tumors can indicate a poor patient prognosis (152, 153). A study of neurofibromas by Yang et al. has shown that Nf1^+/-^ mast cells can secrete TGF-β, thus promoting fibroblast proliferation *via* TGF-β (154). In a human prostate cancer microtissue model, mast cells can release tryptase to enhance CAF-induced transformation of epithelial cell morphology, thus playing an important role in prostate cancer progression (155). In the TME of PDAC, mast cells are essential for tumorigenesis. Mast cells can secrete cytokines IL-13 and tryptase and promote the proliferation of PSCs (156). This suggests that mast cell infiltration and activation can contribute to the formation of dense fibrotic stromal formation characteristics of PDACs and that mast cells can promote PSC proliferation in the TME. Thus, targeting mast cells could be a way to improve PDAC therapy. **Table 1** describes tumorigenic effects of various mediators in TME on the relationship between stromal cells and immune cells.

**Table 1:** Tumorigenic effect of the relationship between stromal cells and immune cells in TME.

Cell type	Mediator	Effect function	Type of Tumor	Ref.
**CAFs** **TEC** **CAA**	MCP-1, SDF-1 production → IL-10↑, IL-12↓	Monocyte recruitment M2-like macrophage differentiation	Breast cancer	(45)
	IL-6, SDF-1 production	M2 macrophage differentiation	Prostate cancer	(50)
	CCL-2 (MCP-1) production → CCR2A/2B pathway activation	Monocyte recruitment for TAM differentiation	Breast cancer	(51, 52)
	IL-6/STAT3 pathway activation	Promotion of MDSC differentiation	Pancreatic cancer	(61)
	IL-6/STAT3 activation → IDO production↑	Induction of DC cell	Hepatocellular carcinoma	(66)
	IL-6, STAT3, PD-L1 signaling pathway	Activation of neutrophil Impairment of T cell function	Hepatocellular carcinoma	(67)
	IL-6, GM-CSF production	Induction of TAM infiltration	Colon cancer	(68)
	IL-6, IL-8 production	Attraction of monocyte recruitment for TAM differentiation, NK cell function inhibition	Colorectal cancer	(70)
	TGF-β production → IL-4↑, IL-10↑, IL-12↓ → PD-L1, HLA-DR↑	M2 macrophage polarization	Hepatocellular carcinoma	(78, 79)
	PD-L2, FASL engagement↑	Induction of CD8^+^ T cell death	Lung cancer	(81)
	CXCL12 (SDF-1)-CXCR4 expression	CD4^+^CD25^+^ T cell proliferation	Breast cancer	(82–84)
	CXCL12 → *via* B7H3, CD73, DPP4↑	Attraction of CD4^+^CD25^+^ T cell, Increase T cell survival, differentiation		
	PD-L1(B7H1), B7DC expression IL-6, CXCL8, TNF, TGFB1, VEGFA↑	Induction of T cell apoptosis and FOXP3^+^ Treg proliferation	Head and neck squamous cancer	(85)
	PGE2 production → inhibition of NK receptor (NKp44, NKp30), perforins, granzymes	Inhibition of NK cell function	Melanoma	(99, 100)
	PGE2 expression, IDO production	Suppression of NK cell activation	Hepatocellular carcinoma	(101, 102)
	Notch1-induced VCAM1 expression	Promotion of Neutrophil infiltration	Ovarian, Lung carcinoma, melanoma	(110)
	FasL production → Fas/FasL death signaling activation	Suppression of CD8^+^ T cell	Glioma	(111)
	TGF-β, IL-10 production, via PD1/PD-L1 pathway	Attenuation of CD8^+^ T cell function	Melanoma	(112)
	CLEVER-1/stabilin-1 production	FOXP3^+^ Treg recruitment	Hepatocellular carcinoma	(114)
	CCL-2 production → IL-1β/CXCL12 activation	Induction of macrophage recruitment	Breast cancer	(117)
	PD-L1 expression	Inhibition of CD8^+^ T cells	Breast cancer	(120)
	IL-8 production	Induction of Neutrophil recruitment	Pancreatic ductal adenocarcinoma	(122)
**MSC**	TGF-β production	Induction of Treg cell	Breast cancer	(125)
	SDF-1/CXCR7 axis	Induction of Breg cell	Non-cancerous	(126)
	TNF-α production → induction PD-L1↑	Suppression of CD8^+^ T cell	Colon cancer	(131)
**Neutrophil**	IL-1β production → MMP-9 activation	EC activation → metastasis ability↑	Pancreatic ductal adenocarcinoma	(132–134)
**Monocyte/** **Macrophage**	OPN production	CAF proliferation → promoting malignancy↑	Hepatocellular carcinoma	(141)
	Wnt7a expression → Wnt/β-catenin signaling	Myofibroblasts differentiation of MSC → fibrosis↑	Non-cancer (Pulmonary fibrosis)	(144)
	miR-155-5p, 221-5p in MDE	TEC proliferation → promoting growth↑	Pancreatic ductal adenocarcinoma	(147, 148)
**NK cell**	VEGF, PIGF production	HUVECs migration, formation↑ → tumor growth↑, angiogenesis↑	Non-small cell lung cancer	(150)
**Mast cell**	TGF-β production	Myofibroblasts differentiation induction, proliferation↑	Neurofibromas	(154)
	Tryptase production	Promoting the transformation of prostate ECs morphology	Prostate cancer	(155)
	IL-13, Tryptase production	Stimulation of PSC proliferation	Pancreatic ductal adenocarcinoma	(156)

CAFs, Cancer-associated fibroblasts; TECs, Tumor-endothelial cells; CAAs, Cancer-associated adipocytes; PSC, Pancreatic stellate cell; TAN, Tumor-associated neutrophil; CM, Conditioned medium; EC, Endothelial cells; FasL, Fibroblast associated ligand; OPN, Osteopontin; MDE, Macrophage-derived exosomes; VEGF, Vascular endothelial growth factor; PIGF, Placental growth factor; HUVEC, Human umbilical vein endothelial cells. ↑, increase; ↓, decrease.

## Anti-Tumorigenic Association Between Stromal Cells and Immune Cells in the TME

### Crosstalk Between Stromal Cells and Immune Cells in the TME Induces Anti-Tumor Immunity

Although numerous studies have suggested that CAFs can exert a tumorigenic effect, other studies have suggested that they are also involved in tumor suppression. According to *Ozdemir*’s study, CAF-depleted tumors are associated with increased CTLA-4 expression with a reduced Teff/Treg cell ratio in the PDAC model. In this study, the cytotoxic Teff/Treg ratio was decreased in the myofibroblast-depleted tumor and associated with a significant elevation in CTLA-4. Thus, CAF depletion can induce immunosuppression, reduce survival, and further accelerate PDAC (157). Suppression of the checkpoint blockade using CAF depletion and anti-CTLA-4 antibody can improve mouse PDAC tumors and increase overall survival (157). CAF depletion has breakthrough efficacy with significant changes in major contributors to cancer development within the TME. McAndrews et al. have demonstrated that depletion of αSMA^+^ CAFs is associated with increased Lgr5^+^ cancer stem cells and the generation of an immunosuppressive TME with increased frequency of Foxp3^+^ Treg cells and suppression of CD8^+^ T cells. Thus, αSMA^+^ CAFs in CRC can promote an anti-tumor effect *via* BMP4/TGF-β signaling (158). Interestingly, infiltrated CD8^+^ T cell accumulation in non-small cell lung cancer with a high proportion of fibroblasts is associated with the expression of CCL19 identified in the lungs and tumors of patients (159). Fibroblasts expressing CCL19 can form perivascular niches and promote the accumulation of CD8^+^ T cells. These results can be proposed as a new marker for immune tumor treatment by targeting CAFs that produce CCL19 (159). A study by Kamata et al. using G12DKRAS and V600EBRAF-driven mouse models that develop lung adenocarcinoma and adenoma has confirmed that stanniocalcin1 secreted by tumor-associated fibroblasts can inhibit TAM differentiation. The secreted stanniocalcin1 can inhibit TAM differentiation by sequestering the binding of glucose regulatory protein 94 (GRP94), an autocrine macrophage differentiation-inducing factor, to the scavenger receptor (160).

A variety of cytokines have been shown to be either pro- or anti-inflammatory depending on the cell type and disease model. IL-33 is an inflammatory cytokine released during necrotic cell death (161). CAFs can release IL-33, induce metastasis *via* EMT, and promote cell migration and invasion. In cancer immune response, IL-33 exhibits both pro-tumoral functions and antitumor functions (162). IL-33 can also induce IL-33 gene expression in HNSCC cells through a positive feedback process (163). The production of IL-33 is responsible for the antitumor response as CD8^+^ T cell infiltration. In a colon cancer model, IL-33 can increase interferon (IFN)-γ production by tumor-invasive CD4^+^ and CD8^+^ T cells. This has been confirmed by the accumulation of infiltrated CD8^+^ T cells, which exerts an antitumor effect (164). These results demonstrate the synergistic increase in IFN-γ, and IL-12 release, along with increases in proliferation, infiltration, and the number of cytotoxic NK cells activated by IL-33 (165). IL-33 administered to a mouse breast cancer model can potently suppress lung metastasis and increase the number of NK cells recruited within the TME (166). Furthermore, it has been shown that NK cell depletion in IL-33/ST2-deficient mice is associated with tumor growth promotion (167).

Interestingly, inhibitor of κB kinase beta (IKKβ)-depletion in intestinal mesenchymal cells (IMCs) can decrease immune cells infiltration and the expression of several pro-inflammatory mediators. Supernatant of IKKβ-depleted IMCs mouse model also shows decreased secretion of chemokine MIP2, cytokines IL-6, TNF, FOX2, and MMP9 in organ culture. Therefore, IKKβ in IMCs of inflammation-associated colorectal cancer might have a tumor-suppressive effect (168).

Several studies have reported that the sirtuin 1 (SIRT1) signaling pathway can regulate vascular inflammation. The role and molecular interaction of SIRT1 and Toll-like receptor 2 (TLR2) in monocyte adhesion to the vascular endothelium have been found to be important. These results suggest potential therapeutic targets for a variety of vascular inflammation, including atherosclerosis (169–171), although these results are not about cancer. Recent studies have shown that extracellular vesicles (EVs) derived from immune cells can perform various roles in immune responses (172). EVs can deliver not only proteins, but also biomolecules such as nucleic acids and lipids. They play a very essential role in the cell-to-cell communication process. Activated CD8^+^ T cells in a mouse model can temporarily release cytotoxic EVs and prevent the progression, invasion, and metastasis of fibroblast stroma-mediated tumors (173).

In platinum-based chemotherapy for ovarian cancer cells, IFN-γ-producing CD8^+^ T cells show altered glutathione (GSH)/cystine metabolism of fibroblasts and reduced fibroblast-mediated platinum resistance (174). Thus, IFN-γ-producing CD8^+^ T cells can eliminate chemoresistance of ovarian tumors and offer a combined treatment method that utilizes immune and stromal cell relationships in cancer treatment. In addition to effector T cells, other immune cells can also influence tumors by controlling fibroblast function. Interestingly, when DCs as potent APCs are fused with CAFs, they can stimulate T cells to attack cancer cells. These DC/CAF fusion cells can produce TNF-α, IL-1β, IL-6, and IL-12p70 and stimulate T cells to produce IFN-α and IFN-γ. T cells activated by the fusion of DCs and CAFs can induce a strong cytotoxic T cell response that has emerged as a new antitumor response through tumor growth inhibition (175). A multiple myeloma study has analyzed the activity of cytokine-stimulated NK cells on tumor-associated endothelial cells. IL15-activated-NK cells can enhance the killing of multiple myeloma patient’s endothelial cells *via* DNAX accessory molecule 1 (DNAM-1) (176). **Table 2** describes anti-tumorigenic effects of various mediators in TME on the relationship between stromal cells and immune cells. It is necessary to understand the complexity between stromal cells and immune cells and carefully consider their targeting and use in drug development to overcome cancer treatment resistance.

**Table 2:** Anti-tumorigenic effect of the relationship between stromal cells and immune cells in TME.

Cell type	Mediator	Effect function	Type of Tumor	Ref.
**CAF** **TEC**	Regulation of CTLA4 expression	Balance of Teff/Treg ratio	Pancreatic adenocarcinoma	(157)
	BMP/TGF-β signaling → Lgr5^+^ CSCs↓	Suppression of FOXP3^+^ Treg Induction of CD8^+^ T cell	Colorectal cancer	(158)
	CCL19 production	Intratumoral accumulation of CD8^+^T cell infiltration↑	Lung carcinoma	(159)
	STC1 production → Inhibition of GRP94 binding on TAM	Inhibition of TAM differentiation	Lung adenocarcinoma Lung adenoma	(160)
	IL-33 production → IL-12↑, IFN-γ↑	Promotion of CD8^+^ T cell infiltration	Colon cancer	(163, 164)
		Induction of cytotoxic NK cell proliferation	Breast cancer	(165–167)
	NF-kb-IKKβ signaling in intestinal mesenchymal cells (IMCs)	Induction of T cell infiltration	Inflammation-associated colorectal cancer	(168)
	SIRT1/TLR2 interaction↑	ECs-monocyte adhesion Inflammation↑	Non-cancer (Vascular inflammation)	(170)
**T cell**	Activated CD8^+^ T cell derived EVs	CAF progression, invasion, and metastasis inhibition	Pancreatic cancer	(173)
	Regulation of GSH/Cystine Metabolism in CAF	Diminished fibroblast mediated platinum resistance	Ovarian cancer	(174)
**DC**	Induction of TNF-α, IL-1β, IL-6, and IL-12p70	Fusion with CAF and induction of T cell stimulation	Hepatoma	(175)
**NK cell**	DNAM-1 activation	Suppression of EC and induction of NK cell cytotoxicity	Multiple myeloma	(176)

CSCs, Cancer stem cells; CCL19, Chemokine (C-C motif) ligand 19; STC1, Stanniocalcin-1, GRP94, Glucose-regulated protein 94; IKK, Inhibitor of nuclear factor-kB kinase; SIRT1, Sirtulin1; TLR2, Toll like receptor 2; EV, Extracellular vesicles; GSH, Glutathione.; DNAM1, DNAX accessory molecule (CD226). ↑, increase; ↓, decrease.

## Conclusions and Prospection

**Figure 1:**
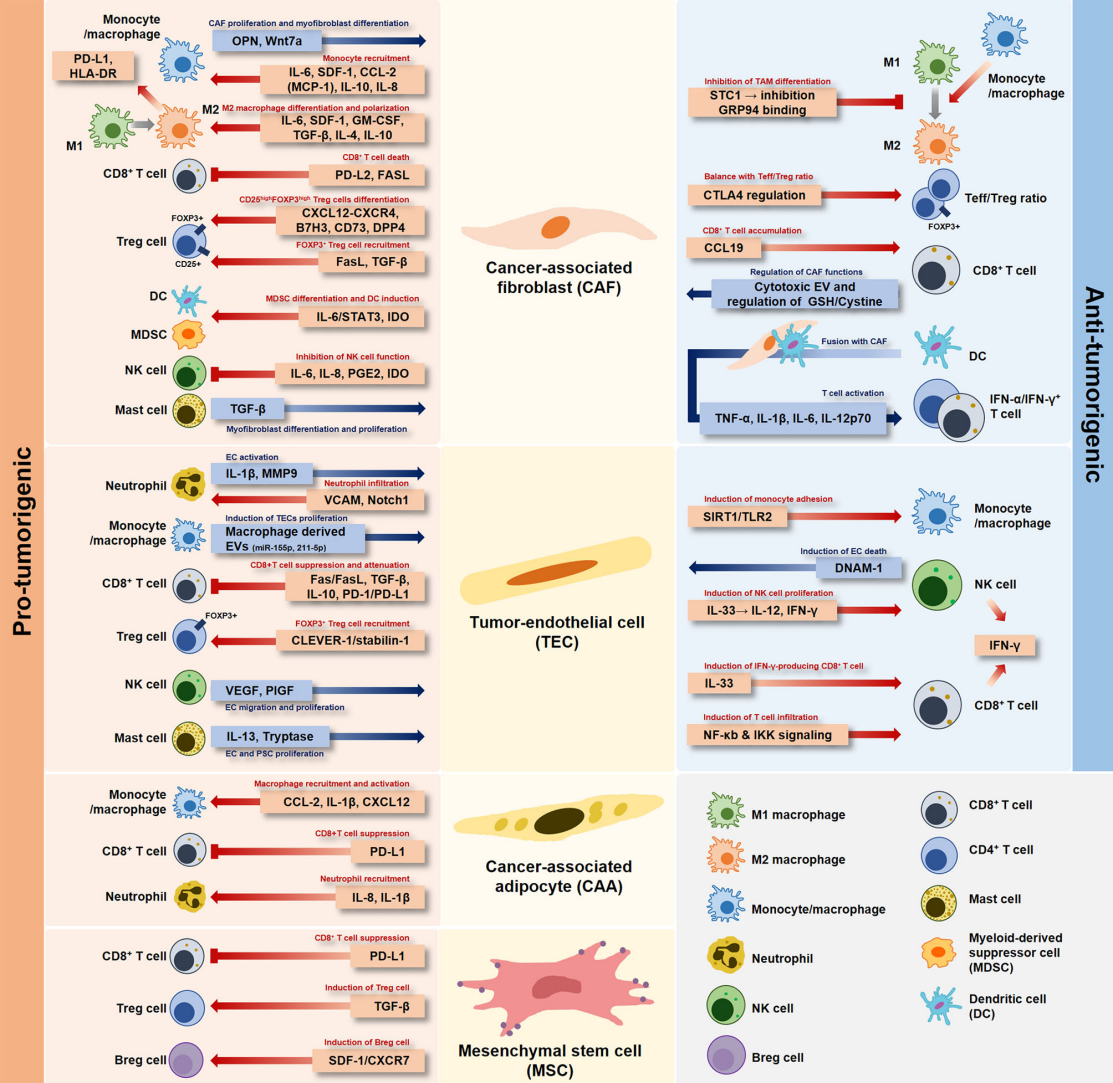
Dual role of stromal cells in the tumor microenvironment. Each stromal cell developed in the tumor-specific microenvironment has a pro-tumorigenic or anti-tumorigenic role depending on the interaction with immune cells.

Early studies have focused on “tumor cells” to explain anticancer drug resistance and progression of tumors by specific signaling mechanisms, rather than heterogenic TME characteristics. Successful cancer treatment strategies need to be developed based on understanding the relationship between stromal cells and immune cells in the TME. Recently, research on cancer cells has continued to transition toward studying the network of stromal cells and inflammatory immune cells in the TME. Ultimately, the organic relationship between stromal cells and immune cells present in the TME not only involves pro-tumorigenic and anti-tumorigenic cells, but also shows a mixed environment of these aspects in the TME (**Figure 1**).

Since the TME is heterogeneous, the relationship between stromal cells and immune cells, which are components of the TME, is complex. However, additional mechanisms that control the interactions between immune cells and stromal cells in the TME remain unknown. Therefore, rather than interpreting a one-way relationship between two cells, it is necessary to understand and approach two-way relationships. The relationship between immune cells and stromal cells in the TME merits further investigation.

In particular, the most interesting aspect of the two orientations proposed in this review is the association of stromal cells with immune cells in controlling tumor formation and development. The network requires more attention when developing drugs that target their relationships. Increasing the understanding of the network between stromal cells and immune cells within the TME will ultimately improve the development and efficacy of cancer therapies. Finally, understanding the complex interactions between stromal cells and immune cells within the TME is necessary to identify potential strategies for cancer treatment.

## Author Contributions

HK contributed to study conception. J-YM, S-HL, and JL performed literature review and analysis and revised the manuscript. J-YM and HK drafted the manuscript, figures, and tables. All authors contributed to the article and approved the submitted version.

## Funding

This research was supported by the National Research Foundation of Korea (NRF) grant funded by the Korean government (NRF-2020R1C1C1003676).

## Conflict of Interest

The authors declare that the research was conducted in the absence of any commercial or financial relationships that could be construed as a potential conflict of interest.

## Publisher’s Note

All claims expressed in this article are solely those of the authors and do not necessarily represent those of their affiliated organizations, or those of the publisher, the editors and the reviewers. Any product that may be evaluated in this article, or claim that may be made by its manufacturer, is not guaranteed or endorsed by the publisher.
